# Trends in physical activity and inactivity amongst US 14–18 year olds by gender, school grade and race, 1993–2003: evidence from the youth risk behavior survey

**DOI:** 10.1186/1471-2458-6-57

**Published:** 2006-03-07

**Authors:** Jean Adams

**Affiliations:** 1 School of Population and Health Sciences, University of Newcastle upon Tyne, Newcastle upon Tyne, UK

## Abstract

**Background:**

Recent increases in the prevalence of adolescent obesity have been widely documented. Whilst there is a common lay perception that the current generation of adolescents is less active than ever before, there is little published data to support this notion. In addition, there is little published data on trends in physical activity in adolescents according to factors such as gender, age and race.

**Methods:**

Data from the US Youth Risk Behavior Survey were used to explore time trends in physical activity (vigorous activity on three or more days in the last week) and inactivity (no vigorous activity in the last week) overall and according to gender, school grade and race amongst US adolescents between 1991 and 2003. Logistic regression was used to assess: the overall change in odds of adolescents being active or inactive per year, the change in odds of adolescents being active or inactive in each survey year compared to the first year for which data was included (1993), and the change in odds of adolescents being active or inactive in each survey year compared to the previous survey year. After analysing data for all individuals combined, separate analyses were performed by gender, school grade and race.

**Results:**

There was evidence of small, but statistically significant, overall trends towards decreased physical activity and increased inactivity over time amongst boys and those in school grades 9 and 10. Whilst few consistent survey to survey trends were seen, there was a significant decrease in the odds of all adolescents, boys and those in school grades 9 and 10 being active between 1993 and 2003 and a significant increase in the odds of the same groups being inactive between 1993 and 2003.

**Conclusion:**

Overall changes in both activity and inactivity were generally small and are unlikely to play a significant role in reported secular trends in overweight and obesity in adolescents.

## Background

Recent increases in the prevalence of adolescent obesity have been widely documented. [1-5]. Whilst there is a common lay perception that the current generation of adolescents is less active than ever before[6], there is little published data to support this notion[1]. Furthermore, whilst there is evidence that physical activity in adolescents varies according to gender, school grade and race, there is little published data on recent trends in physical activity according to these variables. [7-10]. This study reports trends in physical activity and inactivity amongst US adolescents by gender, school grade and race, over the period 1993–2003 using data from the Youth Risk Behavior Survey (YRBS).

## Methods

The YRBS has been conducted biennially since 1991. Participants are recruited via a three stage cluster design and complete a multiple choice questionnaire during school time. Responses are weighted to obtain a representative sample of adolescents in school grades 9–12 in the US in terms of gender, race and school grade. [11-18]. Annual sample sizes are consistently over 10 000[18].

Physical activity and inactivity were determined from the number of days adolescents reported "exercise or sports activities that make you sweat and breathe hard for 20 minutes or more" (vigorous activity) in the last week. As the 20 minute component of this question was not included in the 1991 survey, 1991 data was not included in this analysis. Individuals reporting vigorous activity on three or more days in the last week were defined as physically active[19] and those reporting vigorous activity on no days in the last week were defined as inactive.

As time trends can be considered in a number of different ways, variations in the odds of adolescents being active or inactive between 1993 and 2003 were assessed in three different ways using logistic regression. Firstly, year of survey (1993 = 1, 1995 = 3, 1997 = 5 etc) was entered as a continuous variable to determine the overall change in odds of adolescents being active or inactive per year (not per interval between surveys). Next, year of survey was entered as an ordinal variable to determine the change in odds of adolescents being active or inactive in each survey year compared to the first year for which data was included (1993). Finally, year of survey was entered as an ordinal variable in analyses restricted to adjacent pairs of survey years (1993 and 1995 only, 1995 and 1997 only etc) to determine the change in odds of adolescents being active or inactive in each survey year compared to the previous survey year.

After analysing data for all individuals combined, separate analyses were performed by gender (male or female), school grade (grade 9, 10, 11 or 12) and race (non-Hispanic white, black or African-American, Hispanic white, or other).

Differences in the overall change in odds of adolescents being active or inactive per year according to gender, school grade and race were investigated by introducing interaction terms into the models where survey year was used as a continuous variable and assessing the influence of these terms using Wald tests.

All analyses were performed in Stata v8.0[20] using the stratum, primary sampling units and weights given in the original data sets and the 'svy' set of commands in Stata in order to take account of the complex sample design[21]. Ethical approval was not required for this analysis of publicly available data.

## Results

Table 1 shows the weighted percentage of adolescents who were physically active by survey. Similar data for inactivity are shown in Table 2. In general, boys, those in earlier school grades, and those identifying themselves as non-Hispanic white or Hispanic white tended to be more likely to be physically active and less likely to be inactive.

Tables 3 shows the overall change in odds of adolescents being defined as active per year and in each year compared to 1993. Similar results for the odds of being defined as inactive are shown in Table 4. The overall change in odds per year of adolescents being active or inactive was not statistically significant when all adolescents were considered together. However, when gender, school grade and racial sub-groups were considered, the odds of boys and those in school grades 9 and 10 being active decreased over time (change in odds per year (95% CI) in boys: 0.98 (0.97 to 0.99); school grade 9: 0.98 (0.96 to 0.99); school grade 10: 0.98 (0.96 to 0.99)) whilst the odds of those in the same groups being inactive increased over time (boys: 1.03 (1.01 to 1.05); school grade 9: 1.03 (1.01 to 1.05); school grade 10: 1.02 (1.00 to 1.04)). When the odds of being active or inactive in each year were compared with 1993, all adolescents, boys and those in school grades 9 and 10 were significantly less likely to be active (odds ratio (95% CI) in all adolescents: 0.87 (0.77 to 0.98); boys: 0.79 (0.68 to 0.91); school grade 9: 0.74 (0.60 to 0.92); school grade 10: 0.81 (0.66 to 0.99)) and more likely to be inactive (odds ratio (95% CI) in all adolescents: 1.21 (1.02 to 1.43); boys: 1.41 (1.16 to 1.71); school grade 9: 1.63 (1.27 to 2.09); school grade 10: 1.32 (1.06 to 1.63)) in 2003 compared to 1993. Other scattered statistically significant changes in the odds of being active or inactive were seen, all showing a trend towards decreased likelihood of being active and increased likelihood of being inactive over time.

The odds of adolescents being active or inactive compared to the previous survey year are shown in Table 5 and Table 6 respectively. Only scattered statistically significant change in odds between survey years were seen. In general, these showed a trend towards decreasing likelihood of being active and increasing likelihood of being inactive over time. However, there was a statistically significant decrease in the odds of adolescents who identified their race as neither non-Hispanic white, black or African American, or Hispanic white being inactive between 1997 and 1999 (odds ratio (95% CI): 0.67 (0.45 to 0.99)).

Tests for interaction revealed that the overall change in odds per year of being both active and inactive varied significantly between boys and girls (active: F = 12.40, p < 0.001; inactive: F = 10.85, p = 0.001). Boys became less likely to be active and more likely to be inactive over time than girls. There were no statistically significant interactions between race and year on the odds of adolescents being either active or inactive (active: F = 1.26, p = 0.262, inactive: F = 1.27, p = 0.260) or between school grade and year on the odds of adolescents being active (F = 3.09, p = 0.080). However, there was evidence of an interaction between school grade and year on the odds of adolescents being inactive (F = 4.07, p = 0.045). In particular, those in school grade 9 were significantly less likely to become inactive over time than those in school grade 12.

## Discussion

Using data from six large, representative, samples of US adolescents, this analysis found evidence of small, but statistically significant, overall trends towards decreased physical activity and increased inactivity over time amongst boys and those in school grades 9 and 10 – the same groups where physical activity is generally highest and inactivity lowest. Whilst few consistent survey to survey trends were seen, there was a significant decrease in the odds of all adolescents, boys and those in school grades 9 and 10 being active between 1993 and 2003 and a significant increase in the odds of the same groups being inactive between 1993 and 2003.

The YRBS relies on self reported physical activity data. Concerns have been raised over the validity of such data[22], and it is also possible that validity has altered over time and varies according to any or all of the subgroups investigated here (gender, age and race). Whist such variations may account for the patterns reported here, questionnaire methods may be the only feasible method of collecting information on health related behaviours from such large samples on a frequent basis. In addition, the definition of physical activity used here is only one of a number of such definitions[23]. However, it was the current definition when the YRBS was initiated[19], is evidence-based[24] and is the only definition for which data has been collected in most YRBS surveys.

Although some statistically significant differences in the overall change in odds of being both physically active and inactive per year and in year to year comparisons were found, the magnitude of odds ratios was small in all cases. This suggests that there were only small variations in behaviour across time. Furthermore, examination of the year to year data suggests that variations were not consistent across time. It, therefore, seems unlikely that recent change in physical activity amongst US adolescents play a substantial role in concurrent increases in body weight.

Other data on secular trends in physical activity are not consistent across time period or population – perhaps, at least partly, due to variations in definitions. The percentage of US adults who undertook 'sufficient' physical activity (20 minutes of vigorous activity on three or more days per week or 30 minutes of moderate activity on five or more days per week) increased slightly between 1990 and 1998[25] but stayed constant between 2001 and 2003[26]. In contrast, the proportion of Australian adults who undertook at least 150 minutes of moderate activity per week decreased significantly between 1997 and 2000[27]. Whilst the proportion of English adults who met recommended activity levels increased between 1997 and 2000, this trend was only significant for middle aged and older adults[28]. Amongst children, a recent review concluded that there was only evidence that physical activity in specific contexts such as active transport, organised sport and physical education in school was declining[29]. Other investigations of YRBS data have found no evidence of overall declines in physical activity or enrolment in physical education classes and activity during these classes between 1991 and 2003[10,30]. These inconsistencies in recent data echo the small and inconsistent trends reported here.

## Conclusion

This analysis of data from the YRBS between 1991 and 2003 suggests that whilst there is some evidence of decreased physical activity and increased inactivity amongst US adolescents between 1991 and 2003, overall changes are small and are unlikely to play a substantial role in reported secular trends in overweight and obesity in adolescents.

## Competing interests

The author(s) declare that they have no competing interests.

## Authors' contributions

JA devised the idea for this analysis, obtained and analysed the data and wrote the manuscript.

## Pre-publication history

The pre-publication history for this paper can be accessed here:



## Figures and Tables

**Table 1:** Weighted percentage of adolescents defined as physically active^1 ^by survey year

	**Weighted percentage (95% CI)^2^**					
	**1993**	**1995**	**1997**	**1999**	**2001**	**2003**

**All**	65.8 (64.2 to 67.3)	63.7 (60.2 to 67.1)	63.7 (61.5 to 65.9)	64.7 (62.5 to 66.9)	64.6 (63.0 to 66.2)	62.7 (60.3 to 65.0)
**Gender**						
**Girls**	56.2 (53.7 to 58.6)	52.1 (47.3 to 57.0)	53.5 (49.6 to 57.4)	57.1 (54.6 to 59.5)	57.0 (54.6 to 59.5)	55.1 (52.0 to 58.1)
**Boys**	74.7 (73.0 to 76.4)	74.4 (72.0 to 76.7)	72.3 (70.1 to 74.3)	72.4 (69.0 to 75.5)	72.6 (70.8 to 74.4)	70.0 (67.5 to 72.3)
**School grade**						
**9**	74.5 (71.4 to 77.5)	71.5 (66.0 to 76.5)	72.7 (69.5 to 75.7)	72.5 (67.4 to 77.1)	71.9 (69.1 to 74.6)	68.5 (65.3 to 71.5)
**10**	69.5 (66.6 to 72.2)	69.3 (65.0 to 73.3)	65.9 (63.1 to 68.7)	64.7 (60.8 to 68.4)	67.0 (65.0 to 69.0)	64.9 (61.2 to 68.3)
**11**	62.5 (59.7 to 65.3)	60.3 (54.5 to 65.7)	60.0 (56.2 to 63.8)	58.2 (55.7 to 60.7)	61.3 (58.7 to 63.9)	60.1(57.3 to 62.8)
**12**	57.8 (54.8 to 60.7)	54.9 (51.9 to 57.9)	57.5 (53.6 to 61.4)	61.4 (57.4 to 65.3)	55.5 (52.2 to 58.7)	55.0 (52.4 to 57.6)
**Race**						
**NHW^3^**	67.8 (66.1 to 69.3)	67.0 (62.3 to 71.5)	66.8 (63.5 to 70.0)	67.4 (64.7 to 70.0)	66.5 (64.7 to 68.4)	65.2 (62.0 to 68.3)
**Black^4^**	60.0 (56.0 to 63.9)	53.2 (49.5 to 56.9)	53.9 (50.8 to 57.0)	55.6 (51.2 to 60.0)	59.7 (55.6 to 63.7)	54.9 (51.2 to 58.5)
**HW^5^**	59.4 (54.0 to 64.6)	57.3 (53.6 to 61.0)	60.4 (57.2 to 63.5)	58.8 (53.4 to 64.0)	59.0 (56.0 to 62.0)	58.5 (55.7 to 61.3)
**Other**	65.3 (61.3 to 67.3)	63.5 (57.0 to 67.1)	61.3 (55.2 to 67.0)	64.9 (62.3 to 66.8)	63.6 (59.5 to 67.5)	63.1 (59.7 to 66.4)

^1 ^reported "exercise or sports activities that make you sweat and breathe hard for 20 minutes or more" on three or more days in last week; ^2^95% confidence intervals; ^3^non-Hispanic white; ^4^black or African-American; ^5^Hispanic white

**Table 2:** Weighted percentage of adolescents defined as inactive^1 ^by survey year

	**Weighted percentage (95% CI)^2^**					
	**1993**	**1995**	**1997**	**1999**	**2001**	**2003**

**All**	15.7 (14.6 to 16.8)	16.0 (13.8 to 18.5)	17.6 (15.9 to 19.4)	16.0 (14.5 to 17.7)	15.8 (14.7 to 16.9)	18.3 (16.2 to 20.7)
**Gender**						
**Girls**	20.8 (19.1 to 22.6)	22.0 (18.2 to 26.4)	24.2 (21.2 to 27.6)	20.8 (18.3 to 23.4)	20.0 (18.2 to 21.9)	22.2 (19.3 to 25.3)
**Boys**	10.9 (9.8 to 12.0)	10.5 (9.2 to 11.8)	12.1 (11.1 to 13.2)	11.4 (9.4 to 13.7)	11.3 (10.3 to 12.4)	14.7 (12.7 to 16.8)
**School Grade**						
**9**	9.5 (8.3 to 10.8)	12.3 (9.8 to 15.3)	13.3 (11.5 to 15.3)	12.0 (9.6 to 15.0)	11.2 (9.9 to 12.6)	14.6 (12.2 to 17.4)
**10**	13.4 (11.8 to 15.3)	12.1 (9.8 to 14.8)	16.7 (14.7 to 18.9)	15.2 (12.1 to 18.9)	14.1 (12.5 to 15.8)	17.0 (14.8 to 19.4)
**11**	17.2 (15.5 to 19.0)	18.7 (14.5 to 23.8)	17.9 (15.3 to 20.8)	19.3 (16.5 to 22.4)	18.9 (17.3 to 20.7)	20.2 (17.3 to 23.3)
**12**	21.6 (19.2 to 24.2)	20.4 (16.9 to 24.4)	21.8 (17.8 to 26.3)	19.0 (15.2 to 22.2)	20.6 (18.2 to 23.3)	22.9 (19.6 to 26.7)
**Race**						
**NHW^3^**	14.2 (13.0 to 15.6)	13.3 (11.3 to 15.7)	15.2 (13.0 to 17.7)	14.3 (13.0 to 15.8)	14.4 (13.1 to 15.9)	16.7 (13.9 to 20.0)
**Black^4^**	22.3 (20.2 to 24.6)	25.1 (22.0 to 28.4)	26.0 (22.7 to 29.6)	24.5 (19.3 to 30.5)	19.8 (17.5 to 22.3)	23.8 (21.2 to 26.6)
**HW^5^**	17.1 (15.2 to 19.1)	20.5 (16.6 to 25.0)	17.3 (14.5 to 20.6)	17.8 (14.7 to 21.4)	18.1 (16.4 to 19.9)	20.1 (17.8 to 22.6)
**Other**	15.2 (12.5 to 18.4)	16.5 (11.0 to 23.9)	20.8 (16.6 to 25.7)	14.9 (11.5 to 19.0)	17.8 (15.4 to 20.4)	18.5 (15.6 to 21.8)

^1 ^reported "exercise or sports activities that make you sweat and breathe hard for 20 minutes or more" on no days in last week; ^2^95% confidence intervals; ^3^non-Hispanic white; ^4^black or African-American; ^5^Hispanic white

**Table 3:** Change in odds of adolescents being defined as physically active^1 ^per year and compared to 1993

	**Change in odds per year (95% CI)^2^**	**Odds ratio (95% CI)^2^, compared to 1993**					
		
		**1993**	**1995**	**1997**	**1999**	**2001**	**2003**
**All**	0.99 (0.98 to 1.00)	1.00	0.91 (0.78 to 1.07)	0.92 (0.82 to 1.03)	0.96 (0.85 to 1.07)	0.95 (0.86 to 1.05)	0.87 (0.77 to 0.98)*
**Gender**							
**Girls**	1.01 (0.99 to 1.02)	1.00	0.85 (0.69 to 1.05)	0.90 (0.75 to 1.08)	1.04 (0.90 to 1.19)	1.04 (0.90 to 1.19)	0.96 (0.82 to 1.11)
**Boys**	0.98 (0.97 to 0.99)*	1.00	0.98 (0.85 to 1.14)	0.88 (0.77 to 1.01)	0.88 (0.74 to 1.06)	0.90 (0.79 to 1.02)	0.79 (0.68 to 0.91)*
**School grade**							
**9**	0.98 (0.96 to 0.99)*	1.00	0.86 (0.64 to 1.15)	0.91 (0.73 to 1.13)	0.90 (0.68 to 1.20)	0.88 (0.71 to 1.07)	0.74 (0.60 to 0.92)*
**10**	0.98 (0.96 to 0.99)*	1.00	0.99 (0.79 to 1.25)	0.85 (0.71 to 1.01)	0.80 (0.65 to 0.99)*	0.89 (0.76 to 1.04)	0.81 (0.66 to 0.99)*
**11**	0.99 (0.98 to 1.01)	1.00	0.91 (0.70 to 1.17)	0.90 (0.74 to 1.09)	0.83 (0.72 to 0.97)*	0.95 (0.81 to 1.11)	0.90 (0.77 to 1.06)
**12**	1.00 (0.98 to 1.01)	1.00	0.89 (0.76 to 1.05)	0.99 (0.81 to 1.20)	1.16 (0.95 to 1.42)	0.91 (0.77 to 1.08)	0.89 (0.76 to 1.04)
**Race**							
**NHW^3^**	0.99 (0.98 to 1.00)	1.00	0.97 (0.78 to 1.20)	0.96 (0.82 to 1.13)	0.98 (0.86 to 1.13)	0.95 (0.85 to 1.06)	0.89 (0.77 to 1.04)
**Black^4^**	0.99 (0.98 to 1.01)	1.00	0.76 (0.61 to 0.94)*	0.78 (0.64 to 0.95)	0.83 (0.66 to 1.06)	0.99 (0.78 to 1.24)	0.81 (0.65 to 1.00)
**HW^5^**	1.00 (0.98 to 1.02)	1.00	0.92 (0.71 to 1.19)	1.04 (0.81 to 1.34)	0.97 (0.72 to 1.32)	0.98 (0.77 to 1.26)	0.96 (0.76 to 1.23)
**Other**	1.00 (0.98 to 1.02)	1.00	0.92 (0.68 to 1.26)	0.84 (0.63 to 1.13)	0.98 (0.73 to 1.32)	0.93 (0.73 to 1.18)	0.91 (0.73 to 1.13)

^1 ^reported "exercise or sports activities that make you sweat and breathe hard for 20 minutes or more" on three or more days in last week; ^2^95% confidence intervals; ^3^non-Hispanic white; ^4^black or African-American; ^5^Hispanic white

*p < 0.05

**Table 4:** Change in odds of adolescents being defined as physically inactive^1 ^per year and compared to 1993

	**Change in odds per year (95% CI)^2^**	**Odds ratio (95% CI)^2^, compared to 1993**					
		
		**1993**	**1995**	**1997**	**1999**	**2001**	**2003**
**All**	1.01 (1.00 to 1.03)	1.00	1.03 (0.85 to 1.24)	1.15 (0.99 to 1.32)	1.03 (0.89 to 1.18)	1.01 (0.90 to 1.13)	1.21 (1.02 to 1.43)*
**Gender**							
**Girls**	1.00 (0.98 to 1.02)	1.00	1.08 (0.84 to 1.38)	1.22 (1.00 to 1.49)	1.00 (0.83 to 1.20)	0.95 (0.82 to 1.11)	1.08 (0.89 to 1.32)
**Boys**	1.03 (1.01 to 1.05)*	1.00	0.96 (0.81 to 1.14)	1.13 (0.97 to 1.31)	1.05 (0.83 to 1.33)	1.05 (0.90 to 1.22)	1.41 (1.16 to 1.71)*
							
**9**	1.03 (1.01 to 1.05)*	1.00	1.34 (1.00 to 1.78)*	1.46 (1.18 to 1.81)*	1.30 (0.98 to 1.73)	1.20 (0.99 to 1.46)	1.63 (1.27 to 2.09)*
**10**	1.02 (1.00 to 1.04)*	1.00	0.89 (0.67 to 1.16)	1.29 (1.05 to 1.59)	1.15 (0.86 to 1.55)	1.06 (0.87 to 1.28)	1.32 (1.06 to 1.63)*
**11**	1.02 (1.00 to 1.04)	1.00	1.11 (0.81 to 1.53)	1.05 (0.84 to 1.31)	1.15 (0.92 to 1.44)	1.13 (0.95 to 1.33)	1.22 (0.98 to 1.51)
**12**	1.00 (0.98 to 1.02)	1.00	0.93 (0.71 to 1.21)	1.01 (0.76 to 1.34)	0.85 (0.67 to 1.08)	0.94 (0.76 to 1.16)	1.08 (0.85 to 1.37)
**Race**							
**NHW^3^**	1.02 (1.00 to 1.03)	1.00	0.93 (0.75 to 1.15)	1.08 (0.87 to 1.33)	1.01 (0.86 to 1.17)	1.01 (0.87 to 1.18)	1.21 (0.95 to 1.53)
**Black^4^**	1.00 (0.98 to 1.01)	1.00	1.16 (0.95 to 1.43)	1.22 (0.99 to 1.51)	1.13 (0.82 to 1.56)	0.86 (0.71 to 1.04)	1.09 (0.90 to 1.31)
**HW^5^**	1.01 (0.99 to 1.03)	1.00	1.25 (0.94 to 1.65)	1.02 (0.80 to 1.30)	1.05 (0.81 to 1.37)	1.07 (0.90 to 1.28)	1.22 (1.00 to 1.49)*
**Other**	1.01 (0.98 to 1.04)	1.00	1.10 (0.66 to 1.81)	1.46 (1.03 to 2.07)*	0.98 (0.68 to 1.40)	1.20 (0.91 to 1.59)	1.26 (0.94 to 1.70)

^1 ^reported "exercise or sports activities that make you sweat and breathe hard for 20 minutes or more" on no days in last week; ^2^95% confidence intervals; ^3^non-Hispanic white; ^4^black or African-American; ^5^Hispanic white

*p < 0.05

**Table 5:** Odds of adolescents being defined as physically active^1 ^compared to previous survey year

	**Odds ratio (95% CI)^2^**				
	**1995 cf 1993**	**1997 cf 1995**	**1999 cf 1997**	**2001 cf 1999**	**2003 cf 2001**

**All**	0.91 (0.78 to 1.07)	1.00 (0.85 to 1.19)	1.04 (0.91 to 1.19)	0.99 (0.89 to 1.12)	0.92 (0.82 to 1.03)
**Gender**					
**Girls**	0.85 (0.69 to 1.05)	1.06 (0.83 to 1.35)	1.16 (0.96 to 1.39)	1.00 (0.87 to 1.15)	0.92 (0.79 to 1.08)
**Boys**	0.98 (0.85 to 1.14)	0.90 (0.77 to 1.05)	1.00 (0.83 to 1.21)	1.01 (0.85 to 1.22)	0.88 (0.76 to 1.01)
**School grade**					
**9**	0.86 (0.64 to 1.15)	1.06 (0.79 to 1.42)	0.99 (0.74 to 1.31)	0.97 (0.74 to 1.28)	0.85 (0.70 to 1.03)
**10**	0.99 (0.79 to 1.25)	0.86 (0.68 to 1.07)	0.95 (0.77 to 1.16)	1.11 (0.92 to 1.33)	0.91 (0.76 to 1.08)
**11**	0.91 (0.70 to 1.17)	0.99 (0.75 to 1.30)	0.93 (0.77 to 1.12)	1.14 (0.98 to 1.32)	0.95 (0.81 to 1.11)
**12**	0.89 (0.76 to 1.05)	1.11 (0.92 to 1.35)	1.18 (0.94 to 1.47)	0.78 (0.64 to 0.96)*	0.98 (0.83 to 1.16)
**Race**					
**NHW^3^**	0.97 (0.78 to 1.20)	0.99 (0.77 to 1.27)	1.02 (0.85 to 1.24)	0.96 (0.84 to 1.11)	0.94 (0.81 to 1.10)
**Black^4^**	0.76 (0.61 to 0.94)*	1.03 (0.85 to 1.24)	1.07 (0.87 to 1.32)	1.18 (0.93 to 1.50)	0.82 (0.66 to 1.02)
**HW^5^**	0.92 (0.71 to 1.19)	1.14 (0.93 to 1.38)	0.94 (0.73 to 1.20)	1.01 (0.79 to 1.29)	0.98 (0.83 to 1.16)
**Other**	0.92 (0.68 to 1.26)	0.91 (0.64 to 1.30)	1.17 (0.82 to 1.48)	0.95 (0.70 to 1.28)	0.98 (0.79 to 1.22)

^1 ^reported "exercise or sports activities that make you sweat and breathe hard for 20 minutes or more" on three or more days in last week; ^2^95% confidence intervals; ^3^non-Hispanic white; ^4^black or African-American; ^5^Hispanic white

*p < 0.05

**Table 6:** Odds of adolescents being defined as physically inactive^1 ^compared to previous survey year

	**Weighted percentage (95% CI)^2^**				
	**1995 cf 1993**	**1997 cf 1995**	**1999 cf 1997**	**2001 cf 1999**	**2003 cf 2001**

**All**	1.03 (0.85 to 1.24)	1.12 (0.91 to 1.38)	0.90 (0.76 to 1.06)	0.98 (0.85 to 1.13)	1.19 (1.01 to 1.41)*
**Gender**					
**Girls**	1.08 (0.84 to 1.38)	1.13 (0.85 to 1.51)	0.82 (0.65 to 1.03)	0.95 (0.79 to 1.15)	1.14 (0.93 to 1.40)
**Boys**	0.96 (0.81 to 1.14)	1.18 (1.00 to 1.38)	0.93 (0.74 to 1.17)	1.00 (0.79 to 1.25)	1.35 (1.11 to 1.63)
**School grade**					
**9**	1.34 (1.00 to 1.78)*	1.09 (0.81 to 1.47)	0.89 (0.66 to 1.20)	0.92 (0.69 to 1.22)	1.36 (1.06 to 1.73)
**10**	0.89 (0.67 to 1.16)	1.46 (1.11 to 1.91)*	0.90 (0.66 to 1.21)	0.92 (0.68 to 1.22)	1.25 (1.-2 to 1.53)*
**11**	1.11 (0.81 to 1.53)	0.95 (0.67 to 1.34)	1.10 (0.85 to 1.42)	0.98 (0.79 to 1.21)	1.08 (0.88 to 1.34)
**12**	0.93 (0.71 to 1.21)	1.09 (0.78 to 1.51)	0.84 (0.62 to 1.15)	1.11 (0.87 to 1.42)	1.15 (0.89 to 1.47)
**Race**					
**NHW^3^**	0.93 (0.75 to 1.15)	1.16 (0.90 to 1.51)	0.93 (0.75 to 1.15)	1.01 (0.86 to 1.18)	1.19 (0.94 to 1.51)
**Black^4^**	1.16 (0.95 to 1.43)	1.05 (0.83 to 1.34)	0.92 (0.65 to 1.30)	0.76 (0.55 to 1.06)	1.27 (1.03 to 1.55)
**HW^5^**	1.25 (0.94 to 1.65)	0.82 (0.59 to 1.13)	1.03 (0.76 to 1.40)	1.02 (0.79 to 1.31)	1.14 (0.95 to 1.37)
**Other**	1.10 (0.66 to 1.81)	1.33 (0.79 to 2.25)	0.67 (0.45 to 0.99)*	1.23 (0.88 to 1.72)	1.05 (0.81 to 1.37)

^1 ^reported "exercise or sports activities that make you sweat and breathe hard for 20 minutes or more" on no days in last week; ^2^95% confidence intervals; ^3^non-Hispanic white; ^4^black or African-American; ^5^Hispanic white

*p < 0.05
